# Development of IFN-*γ* resistance is associated with attenuation of SOCS genes induction and constitutive expression of SOCS 3 in melanoma cells

**DOI:** 10.1038/sj.bjc.6603849

**Published:** 2007-06-19

**Authors:** M Fojtova, V Boudny, A Kovarik, L Lauerova, L Adamkova, K Souckova, J Jarkovsky, J Kovarik

**Affiliations:** 1 Institute of Biophysics, Academy of Sciences of the Czech Republic v.v.i., Kralovopolska 135, 612 65 Brno, Czech Republic; 2 Department of Experimental Oncology, Masaryk Memorial Cancer Institute, Zluty kopec 7, 656 53 Brno, Czech Republic; 3 Faculty of Medicine and Science, Institute of Biostatistics and Analyses, Masaryk University, Kamenice 126/3, 625 00 Brno, Czech Republic

**Keywords:** SOCS proteins, interferon resistance, STAT phosphorylation, malignant melanoma

## Abstract

The resistance to interferons (IFNs) limits their anticancer therapeutic efficacy. Here we studied the evolution of an IFN-resistant state *in vitro* using melanoma cell lines. We found that the cells became less sensitive to antiproliferative effect of IFN-*γ* after prolonged cultivation enabling us to isolate sensitive and resistant subclones of the parental line. We investigated transcription of signal transducer and activator of transcription (STAT) 1–6 and suppressor of cytokine signalling (SOCS) 1–3 genes, and phosphorylation of STAT 1 protein. The resistant subline (termed WM 1158R) differed from the sensitive subline (WM 1158S) by a constitutive expression of SOCS 3, lack or weak SOCS 1–3 activation following IFN-*γ*, and short duration of cytokine activatory signal. Similar correlations were observed in additional melanoma lines differing in IFN sensitivities. At the protein level, IFN-*γ* induced strong and prolonged STAT 1 activation at serine 727 (S727) in WM 1158R while in WM 1158S cells phosphorylation of this amino acid was much less pronounced. On the other hand, phosphorylation of tyrosine 701 (Y701) was stimulated regardless of the sensitivity phenotype. In conclusion, constitutive expression of SOCS 3 is correlated with attenuation of its induction following IFN treatment. These results suggest that progression of melanoma cells from IFN sensitivity to IFN insensitivity associates with changes in SOCS expression.

It has been well established that interferons (IFNs) can inhibit the growth and proliferation of a wide variety of tumour cells. Their action is generally cytostatic rather than cytotoxic, and inhibition of progression through cell cycle phases has been shown. Therapeutic utility of IFN administration has been primarily demonstrated in some leukaemia and from solid type of malignancies in renal cell carcinoma, Kaposi's sarcoma and malignant melanoma –(for review see Lens and Dawes, 2002). However, the effectiveness of the treatment is often unsatisfactory probably due to *per se* resistance of some tumour cell subclones and/or acquired resistance to IFN biological effects.

Biologic activities of IFNs are mediated upon interaction with specific cell surface receptors and activation of intracellular signalling cascades (Ransohoff, 1998). Janus kinase (JAK)/signal transducer and activator of transcription (STAT) proteins play a key role in IFN-induced signalling that consequently modulate essential cellular functions such as growth, differentiation and apoptosis (Brierley and Fish, 2005). STAT 1 is certainly needed for transmission of IFN-*γ* signals (Durbin *et al*, 1996), but the role for other STATs including STAT 3 (Ramana *et al*, 2005) is emerging as well. Recent recognition of JAK/STAT signalling pathways as inevitable molecular mechanism of various cellular effects of IFNs has elicited a considerable effort to elucidate the role of their components in cancer (Calo *et al*, 2003). In malignant melanoma, some studies demonstrated reduced STAT 1 activity in IFN-resistant cell lines pointing to defects in the JAK/STAT pathways (Wong *et al*, 1997; Pansky *et al*, 2000). Alterations occurred at both protein and RNA levels and at post-translational phosphorylation modifications. While most melanoma cell lines displayed normal Y701 phosphorylation response to IFN-*γ* treatment, phosphorylation block at S727 appeared to be more frequent (Kovarik *et al*, 2003). On the other hand, variable STAT 1 levels without apparent correlation with cell sensitivity towards IFNs were also reported (Chawla-Sarkar *et al*, 2002; Jackson *et al*, 2003), and in a clinical study, upregulated STAT 1 phosphorylation was found at high frequency in patients with poor prognosis (Boudny *et al*, 2003). In addition to STAT 1, defects in STAT 5 regulation have recently been shown to contribute to IFN resistance in melanoma cells (Wellbrock *et al*, 2005). It seems that involvement of STAT molecules in oncogenesis may be a rather complex phenomenon.

The duration of STAT function is tightly controlled by several families of negative regulators including a family of classical feedback loop regulators – suppressors of cytokine signalling (SOCSs). Up to now, eight members of these cytokine-induced family have been identified that attenuate or inhibit cytokine growth factor signal –(for review see Hilton, 1999; Fujimoto and Naka, 2003). Out of these, SOCS 1 (Alexander *et al*, 1999) and SOCS 3 (Ramana *et al*, 2005) appear to be critical inhibitors of IFN-*γ* signalling. In tumours, dysfunction of SOCS molecules can cause hyper-responsiveness to cytokines and growth factors and could contribute to the development and/or progression of malignant tumours. For example, silencing of SOCS 1 and/or SOCS 3 genes by methylation of promoter has been correlated with the loss of growth control inhibition in lung (Yoshikawa *et al*, 2001), pancreatic (Komazaki *et al*, 2004), breast and ovarian (Sutherland *et al*, 2004) carcinomas. Similar epigenetic block apparently affected SOCS 3 gene expression in lung (He *et al*, 2003) and head and neck carcinomas (Weber *et al*, 2005). These studies indicated that aberrant silencing could be a cause for constitutive activation of JAK/STAT pathway in cancer cells. However, other studies also indicated that malignant cells compared to their normal counterparts express SOCS genes constitutively (Sakai *et al*, 2002; Li *et al*, 2004; Evans *et al*, 2007) and forced expression of SOCS transgene often conferred resistance to IFN. Constitutive expression can potentially hamper immunotherapy by inactivating cytokine signals. Clearly, many uncertainties remain on the function of SOCS in tumourigenesis.

Our previous screening of melanoma cell lines showed that constitutive expression of SOCS 3 was generally low (Kovarik *et al*, 2005). Nevertheless, few lines showed relatively high level of SOCS 3 expression at both RNA and protein levels. No attempts were made to correlate expression levels with IFN sensitivity. We now aimed to investigate the relationship between IFN sensitivity and expression of STAT and SOCS genes. To reduce the variability caused by variation in genetic and cell type background, we have used two sublines derived from the parental human malignant melanoma WM 1158 line, differing in the sensitivity towards IFN-*γ*.

## MATERIALS AND METHODS

### Cell cultures

Melanoma WM 1158, WM 9, WM 39, WM 1552C, 1205 Lu cell lines (Wistar Institute, Philadelphia, PA, USA) were grown in Dulbecco's modified Eagle's medium (GIBCO, USA) supplemented with 2 g l^−1^ of sodium bicarbonate, L-glutamine, insulin, antibiotics and 10% of fetal bovine serum. Cells were cultured in the incubator with 5% CO_2_ in humidified atmosphere. Human epidermal melanocytes (manufacture code C-002-5C) isolated from lightly pigmented neonatal foreskin were purchased from Cascade Biologics, USA. The cells were grown in the medium 254 supplemented with human melanocyte growth supplement (both Cascade Biologics, USA). The cells were obtained at the end of the secondary culture stage and have passed three to six passages until used for experiments.

### Growth inhibition assay and statistical analyses

**Figure 1:**
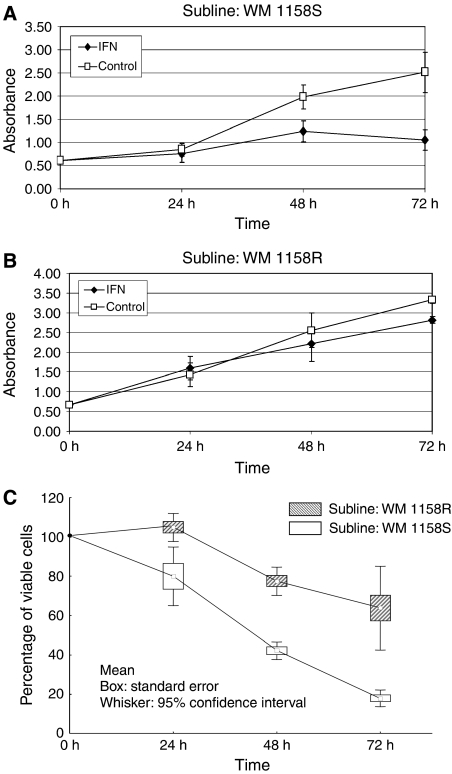
Antiproliferative effect of IFN-*γ* in WM 1158S and WM 1158R sublines measured by WST-1 colourimetric test. (**A** and **B**) The growth is expressed as the amount of viable cells in treated and untreated samples. Each value represents a mean from three independent experiments. Error bars indicate s.d. (**C**) The cell viability normalised to untreated controls. The differences between WM 1158S and WM 1158R sublines were statistically significant (*P*<0.01) for all time intervals (24, 48 and 72 h).

Cells were seeded into 96-well microplates at a density of 2000 cells per well. One day after seeding, the medium was replaced by medium containing IFN-*γ* (Sigma, Saint Louis, MO, USA) at concentrations of 50, 100, and 200 ng ml^−1^. After 24, 48 and 72 h intervals of treatment, WST-1 colourimetric assay (Roche, Mannheim, Germany) was performed according to the manufacturer's protocol. The absorbance was measured in three parallel wells. The growth inhibition (cytotoxicity) was evaluated in at least three independent experiments and expressed as the amount of viable cells in treated and untreated samples (Figure 1A and B) or as the cell viability normalised to untreated controls (Figure 1C). The data were statistically analyzed employing a parametric *t*-test.

### Western blot analyses

The cells were harvested and lysed according to standard procedures (Boudny *et al*, 2003). The protein content in whole-cell extracts was determined by Bradford assay (Bio-Rad, Munich, Germany). Approximately, 20 *μ*g of total proteins were separated by sodium dodecyl sulphate–polyacrylamide gel electrophoresis (10% gels) and the proteins were transferred to nitrocellulose membrane (Bio-Rad, Hercules, CA, USA). Phosphorylated STAT proteins were visualised after immunoprecipitation as described in Kovarik *et al* (2003). After the primary antibody binding reaction, the blots were incubated with either anti-mouse or anti-rabbit horseradish peroxidase-conjugated secondary antibody (Amersham Biosciences, Little Chalfont, Buckinghamshire, UK) and developed using the enhanced chemiluminiscence detection system (Amersham) according to manufacturer's instructions.

For the detection of STAT 1 phosphoforms, commercial STAT 1 anti-pY701 rabbit polyclonal antibody (Cell Signaling Technology, Danvers, MA, USA) and home-developed anti-pS727 mouse monoclonal antibody (pSM1, Kovarik *et al*, 2003) were used. Total STAT 1 protein levels were assayed by rabbit polyclonal antiserum against C-terminal domain of STAT 1 raised in author's lab (S1C).

### RNA analyses

Total RNAs were isolated from cells treated with 50 ng ml^−1^ of IFN-*γ* for various intervals using RNAeasy kit (Qiagen, Hilden, Germany). Each sample was extensively treated with DNaseI. The corresponding cDNAs were prepared by reverse transcription using Superscript II polymerase (Invitrogen, Carlsbad, CA, USA). The cDNAs were amplified using a multigene 12-well format strip (SuperArray, Biosci Co., Frederick, MD, USA) containing sets of primers for seven *STAT*s, four *SOCS*s and *GAPDH* genes. The PCR was carried out in a MJ Research thermocycler according to the manufacturer's recommendation using 30 cycles at a maximum to keep the reaction in a linear phase. The 5–10 *μ*l aliquots were loaded on a 2% agarose gel. The sizes of bands were estimated from size markers and found to fit with expected sizes of amplicons. The ethidium bromide fluorescent signals were scanned by a CCD camera (Ultralum) and quantified (Ultraquant).

In some cases, the reverse transcriptase (RT)–PCR results were verified by Northern blot using SOCS 1 (Hebenstreit *et al*, 2005) and SOCS 3 (Masuhara *et al*, 1997) probes as described in Kovarik *et al* (2005).

## RESULTS

### Differential sensitivity of WM 1158 sublines to interferon-*γ*

Upon continuous cultivation of WM 1158 melanoma cells, we observed gradual decrease of sensitivity towards IFN-*γ*. Repeated cloning resulted in the isolation of resistant (WM 1158R) and sensitive (WM 1158S) sublines. While IFN-*γ* retarded the growth of WM 1158S by 80–90%, the growth of WM 1158R cells was significantly less inhibited (Figure 1 and Table 1). The growth properties of both sublines in the absence of IFN-*γ* were similar (Figure 1A and B) as well as cell morphology (data not shown). The antiproliferative effect of IFN was further studied in several additional melanoma lines (Table 1). These cells can be roughly categorised into high (WM 1158S, WM 39, WM 1552C), medium (WM 1158R) and low (WM 9, 1205 Lu) sensitive ones. The low sensitivity of WM 9 line towards IFN-*γ* was in a good agreement with that previously published (Kortylewski *et al*, 2004).

### Expression profiles before interferon-*γ* treatment

**Figure 2:**
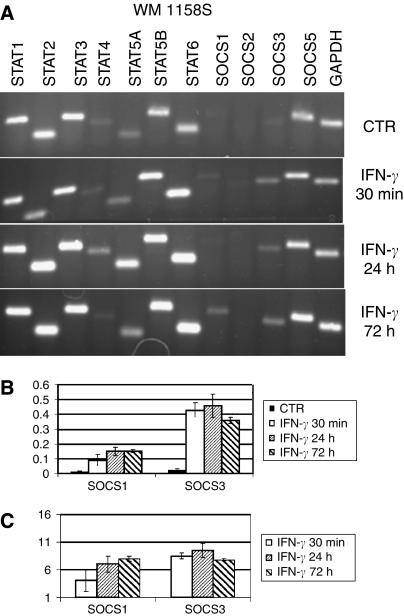
Analysis of STAT and SOCS transcripts in the sensitive WM 1158S subline. (**A**) Total RNAs were prepared from cells treated with IFN-*γ* for 30 min, 24 and 72 h and from non-treated controls. The DNA products of RT–PCRs were separated on 2% agarose gels. (**B**) Transcription is expressed as a SOCS/GAPDH ratio or (**C**) fold increase over basal level. Means of three independent experiments are shown.

**Figure 3:**
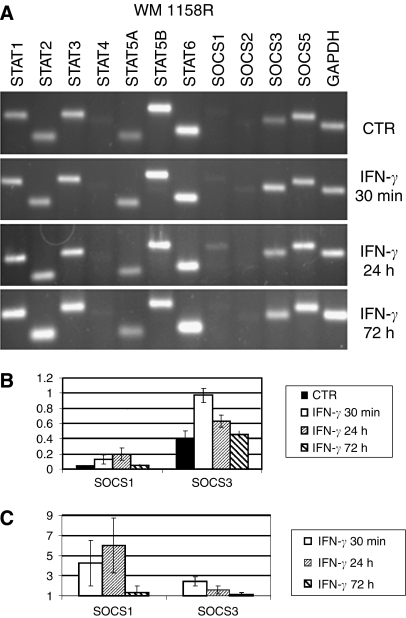
Analysis of STAT and SOCS transcripts in the resistant WM 1158R subline. The conditions and experimental set-up were as in Figure 2.

**Figure 4:**
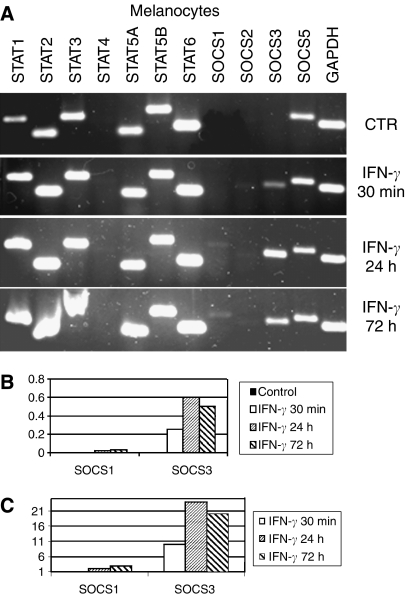
Analysis of STAT and SOCS transcripts in normal human melanocytes. Description of lanes and panels is as in Figure 2. Results of a single experiment are shown.

Reverse transcriptase–PCRs were carried out in 12-well strips allowing simultaneous expression analysis of STAT 1–6, SOCS 1–3 and SOCS 5 genes (Figures 2A, 3A and 4A, upper panels). As a reference, each strip contained primers amplifying a constitutively expressed *GAPDH* gene. Isolated RNAs were reverse transcribed and corresponding cDNAs amplified using specific primers. The first seven lanes in each strip show amplification products obtained with primers specific for various STATs. Most STATs were amplified generating strong bands in all cell lines suggesting their constitutive expression. However, there were differences in the intensities of individual bands. For example, STAT 4 signal was weak and even missing (in melanocytes) compared to other STATs. The STAT 5B band was consistently stronger than that of STAT 5A.

The SOCS primers were designed to allow detection of SOCS 1, 2, 3, and 5 transcripts. The bands corresponding to SOCS 1, 2, and 3 transcripts were hardly visible in WM 1158S (Figure 2A), indicating that expression of most SOCSs was low or negligible before IFN treatment. In contrast, WM 1158R showed relatively strong SOCS 3 signals (Figure 3A) arguing for a constitutive expression of SOCS 3 in these cells. To quantify transcript levels, we measured fluorescence intensities of individual bands and expressed data as the SOCS 3/GAPDH ratio (Figures 2B, 3B and Table 1). RT–PCR analysis of SOCSs RNA was further carried out in additional melanoma cell lines (Table 1) and in human melanocytes (Figure 4). Relatively strong SOCS 3 signals were observed in both IFN-resistant WM 9 and 1205 Lu lines while low constitutive expression displayed relatively sensitive WM 39 and WM 1552C (Table 1) and the melanocytes (Figure 4A). Compared to SOCS 3, the SOCS 1 signals were barely detectable except of 1205 Lu (data not shown). Abundant SOCS 5 transcripts were detected in all cell lines (exemplified in Figures 2A, 3A and 4A).

### Expression profiles after IFN-*γ* treatment

After the IFN-*γ* treatment, there were no material changes in the STAT transcripts profiles. In melanocytes, there was a moderate (1.5-fold) increase of STAT 1 (Figure 4) while no increase was observed in melanoma lines (Figures 2, 3). However, IFN-*γ* slightly elevated STAT 5 and STAT 6 signals in melanoma cells.

Significant qualitative changes occurred in a spectrum of SOCS-specific bands after the IFN treatment (Figures 2, 3 and 4). The most prominent changes involved SOCS 3 and SOCS 1 transcripts. SOCS 3 expression was significantly induced in WM 1158S subline (Figure 2), WM 1552C and WM 39 cells (Table 1) as well as in normal melanocytes (Figure 4). However, no or weak SOCS 3 signals were detected in WM 9 and 1205 Lu cells (Table 1) and WM 1158R subline (Figure 3). The induction levels were expressed as a ratio of normalised signals in IFN-treated and -non-treated cells (Figures 2C, 3C and 4C, Table 1). Detailed kinetic analysis (Figures 2, 3 and 4) revealed that in both WM 1158S and melanocytes the SOCS 3 signal peaked within 24 h following IFN-*γ* treatment while in WM 1158R the faint induction was time-limited and reached its maximum in a 30 min interval. After 72 h, there was no apparent difference between non-treated and treated WM 1158R cells, whereas WM 1158S still retained elevated SOCS 3 expression.

Intensities of the SOCS 1 bands were generally much lower than those of SOCS 3. Nevertheless, it is evident that SOCS 1 bands were visible after IFN-*γ* in both WM 1158S and WM 1158R and melanocytes. Compared to SOCS 3, the induction was shifted to a longer exposure time. IFN treatment had no apparent effect on SOCS 2 RNA levels.

### STAT 1 phosphorylation

**Figure 5:**
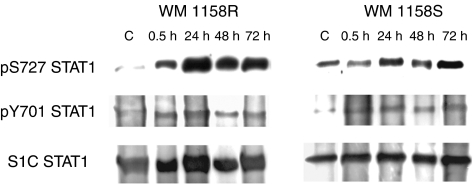
STAT 1 phosphorylation levels in both IFN-resistant and IFN-sensitive WM 1158 sublines. The proteins were extracted from the same cellular pool as used for RNA analysis and analysed by Western blot. All experiments were performed in triplicates.

Several studies indicated the importance of activated (phosphorylated) STAT 1 molecules in IFN resistance. We explored STAT 1 phosphorylation status in cell extracts of sensitive and resistant sublines derived from WM 1158 melanoma cells isolated from different time intervals of IFN treatment (Figure 5). In the resistant WM 1158R subline, the signal corresponding to phosphorylated S727 isoform sharply increased within 30 min after the induction with IFN-*γ* and remained stable thereafter. In contrast, the sensitive WM 1158S cells showed relatively uniform pS727 bands, not significantly influenced by IFN treatment. Both sublines exhibited increased and stable pY701 signals in the presence of IFN-*γ*. STAT 1 levels were not significantly influenced by IFN treatment as revealed by staining of blots with an antibody recognising a primary determinant (Figure 5, bottom panels). Similar STAT 1 phosphorylation results were also observed in additional WM 9 and WM 39 melanoma cell lines as well as in melanocytes (data not shown).

## DISCUSSION

### Development of interferon resistance over cell culture passages

In the present study, we have isolated and characterised interferon-sensitive and -insensitive sublines of a parental melanoma line. Development of cytokine resistance is a known complex phenomenon linked to progression from early to advanced stages of malignant melanoma (Lu *et al*, 1993). It could be a result of selection of pre-existing resistant cells in parental tumour or alteration of signal transduction pathways as a result of genetic/epigenetic cellular changes. Our data do not allow to distinguish between both possibilities. Interestingly, similar switch towards IFN-*γ*-resistant phenotype was previously reported in cervical carcinoma cells (Lee *et al*, 1999), suggesting that emergence of IFN-resistant phenotype might be a general feature of cells that have passed multiple divisions *in vitro* and possibly *in vivo* as well. However, while in their experiments the sensitivity change was correlated with the loss of STAT 1 expression, in our system STAT 1 continued to be expressed and faithfully phosphorylated at tyrosine residues at least. Perhaps there might be multiple alternative pathways influencing antiproliferative potential of IFNs.

### Expression profiles of SOCSs in melanoma cells

The SOCS gene products are known STAT-induced negative regulators of STATs phosphorylation. We took advantage of the availability of sensitive and insensitive derivatives of WM 1158 line to study changes in SOCS expression patterns. In this way, we reduced variation originating from different parental origin (tumour stage, histological type and patient individuality) of cell lines. Out of four SOCS genes investigated, SOCS 3 and SOCS 1 appeared to be the best responders while SOCS 2 and SOCS 5 were insensitive to IFN-*γ* challenge (Figures 2, 3 and 4). The absence of SOCS 5 induction fits with the current view that SOCS 5 is not a classical feedback regulator of cytokine signalling (Fujimoto and Naka, 2003). The induction of SOCS 3 transcripts remarkably differed between the WM 1158 sublines: while the sensitive subline showed in average 7.5-fold induction of SOCS 3, there was only marginal transcript increase in its IFN-resistant variant (Table 1). The differences were less pronounced for SOCS 1, and the induction of this gene also showed higher level of variation (Figures 2, 3). The kinetic experiments showed that the sensitive subline maintained elevated SOCS 1 and SOCS 3 mRNA levels for >72 h following IFN-*γ* challenge while both transcripts were attenuated rapidly to basal levels in the resistant WM 1158R. Significantly, duration of elevated SOCSs expression correlated with the growth inhibition that was most pronounced within the 24–72 h interval (Figure 1). These results indicated differences in the extent and dynamics of SOCS 1 and SOCS 3 IFN-mediated activation between sensitive and resistant sublines. Interestingly, a good correlation between constitutive SOCS 3 expression, lack of its inducibility and IFN cell resistance was obtained in additional melanoma cell lines (Table 1). The strong upregulation of SOCS 3 following IFN-*γ* could be a specific feature of melanoma lineages because epithelial breast cancer cells showed its downregulation (Evans *et al*, 2007) or variable expression (Souckova, unpublished data). However, in breast cancer cells SOCS 1 was strongly activated while its induction was rather weak in melanoma lines used. It is likely that SOCS activation pathways operate differently in diverse cell and tissue types.

In general, the cell sensitivity to interferon was better correlated with inducibility of SOCS 3 than that of SOCS 1. The question arises as to the possible cause of differential activation of these genes. Epigenetic changes in the course of prolonged cultivation of cells have been well described (Chow and Rubin, 2000; Fojtova *et al*, 2003) and both SOCS genes were shown to be inactivated by methylation of their promoters (He *et al*, 2003). However, we consider this possibility unlikely since the IFN-insensitive WM 1158R subline expressed SOCS 3, suggesting that the promoter is transcriptionally active. Although highly specific methylation of an IFN–responsive element in the promoter cannot be entirely excluded, we favour the hypothesis that constitutive SOCS 3 expression could counteract the transactivatory signals delivered by IFN-activated STATs. In support, three IFN-insensitive WM 1158R, WM 9 and 1205 Lu melanoma lines had high basal expression of SOCS 3 compared to sensitive cells. Furthermore, breast cancer (Evans *et al*, 2007) and leukaemia cells (Sakai *et al*, 2002) showed elevated constitutive expression of SOCS 1 and SOCS 3 and the resistance to proinflammatory cytokines including IFN-*γ*. Perhaps IFN sensitivity of a SOCS 3 promoter is reduced due to aberrant expression of an activatory transcription factor(s). In this context, aberrant activation of tissue-specific promoters has been observed in cancers (Kovarik *et al*, 1993).

### Relation between SOCSs induction and STAT 1 phosphorylation

Since STAT 1 is one of the major targets of phosphorylation elicited by IFN-*γ*, we have investigated relationship between STAT 1 phosphorylation at S727 and Y701 residues and SOCS induction. Our semi-quantitative RT–PCR failed to reveal significant differences in STAT 1 levels (both constitutive and induced), suggesting that amounts of gene products were not markedly influenced by IFN (Lesinski *et al*, 2005). At the protein modification level, IFN-*γ* stimulated phosphorylation of Y701 residues in both sensitive and insensitive cells consistent with previous studies that demonstrated lack or weak correlation between Y701 STAT 1 phosphorylation and cell sensitivity to cytokine stimuli (Chawla-Sarkar *et al*, 2002; Kortylewski *et al*, 2004). Surprisingly, the differences in IFN sensitivity were best reflected by the second most commonly phosphorylated site, the S727 residue: while the IFN-sensitive WM 1158S cells did not show marked increase of S727 phosphorylation following IFN-*γ* treatment, there was extensive and prolonged phosphorylation of this amino acid in the insensitive subline. Considering that SOCS 3 regulator was more strongly activated in the IFN-sensitive subline, it is tempting to speculate that phosphorylation of S727 residues was specifically blocked by this molecule. In this context, S727-phosphorylated STAT 1 provided apoptotic resistance for Wilms tumour cells (Timofeeva *et al*, 2006). Although phosphorylation at Y701 was shown to be sufficient for transactivatory capacity of STAT 1 molecule (Shuai *et al*, 1993), S727 phosphorylation seems to be an important modulator of target specificity in haemopoetic cells (Kovarik *et al*, 2001). Perhaps high levels of STAT 1 phosphorylation at S727 might activate specific genes involved in IFN resistance.

### Comparison of normal versus malignant cells

In normal human melanocytes, SOCS 3 appeared to be strongly induced following IFN-*γ* while its basal levels were low or negligible. The induction was quite stable and only marginal decrease occurred within the 72 h treatment interval. In this aspect, there were apparent similarities between normal cells and the IFN-sensitive WM 1158S melanoma subline. However, melanocytes differed from malignant cells in no or marginal SOCS 1 expression. This is congruent with another study showing low levels of SOCS 1 in melanocytes compared to melanoma cell lines (Li *et al*, 2004). However, in their study the lack of SOCS 1 expression occurred at the protein but not at the RNA level. Our failure to detect SOCS 1 in melanocytes might be explained by differences in the sensitivity of the RT–PCR assay (e.g., variable number of cycles), and perhaps by other factors.

### Conclusions and further directions

In conclusion, prolonged maintenance of melanoma cells in cell culture may lead to reduction of their sensitivity to IFN-*γ*. At the molecular level, this process is associated with increased constitutive expression of SOCS 3 whose levels are no longer or marginally influenced by IFN signals. Our data suggest that changes in the SOCS 3 expression are tightly bound with the progression of melanoma cells from IFN-sensitive to IFN-resistant phenotype and may account for a growth advantage of melanoma *in vivo* at its advanced stages. In the future, it will be interesting to analyse the expression of various SOCSs in clinical samples to correlate their expression profiles with patients' responsiveness to IFN-based immunotherapy and disease outcome.

## Figures and Tables

**Table 1:** Relation between cell sensitivity to IFN-*γ*, SOCS 3 mRNA levels and SOCS 3 inducibility

**Line**	**IFN**-*γ* **resistance^a^**	**SOCS 3 constitutive expression^b^**	**SOCS 3 induction (fold increase)^c^**
1205 Lu	90–100	0.7	1.0
WM 9	86–98	0.5	1.3
WM 1158R	68–80	0.4	2.4
WM 39	28–38	0.03	9.5
WM 1552C	39–43	0.03	12.7
WM 1158S	10–20	0.05	7.5

Abbreviations: IFN=interferon; SOCS=suppressors of cytokine signalling.

a Expressed as a percentage of viable cells after the 72 h time interval of IFN-*γ* treatment as related to untreated controls. Range of cytotoxicity values from at least three parallel experiments.

b Assayed by RT–PCR as described in Figures 2 and 3. Fluorescent signals were normalized to GAPDH. Means of at least two independent experiments are shown.

c Fold increase over the non-treated control after 30 min of IFN-*γ* treatment.
